# Independent and Combined Effects of Exercise and Vitamin D on Muscle Morphology, Function and Falls in the Elderly

**DOI:** 10.3390/nu2091005

**Published:** 2010-09-16

**Authors:** Robin M. Daly

**Affiliations:** Department of Medicine (RMH/WH), The University of Melbourne, Western Hospital, Melbourne 3011, Australia; Email: rdaly@unimelb.edu.au; Tel.: +61-3-8345-6924; Fax: +61-3-9318-1157

**Keywords:** vitamin D, resistance training, sarcopenia, falls, older adults, interaction

## Abstract

Regular exercise, particularly progressive resistance training (PRT), is recognized as one of the most effective strategies to prevent age-related muscle loss (sarcopenia), but its effects on muscle function are mixed. However, emerging data indicates that high velocity PRT (fast concentric muscle contractions) is more effective for improving functional outcomes than traditional PRT. In terms of falls prevention, high-challenging balance training programs appear to be most effective. There is also compelling evidence that supplemental vitamin D is an effective therapeutic option for falls prevention. The findings from a recent meta-analysis revealed that supplemental vitamin D at a dose of at least 700–1,000 IU/d or an achieved serum 25(OH)D level of at least 60 nmol/L was associated with reduced falls risk among older individuals. Based on these findings, it is possible that the combination of exercise and vitamin D could have a synergistic effect on muscle morphology and function, particularly since both interventions have been shown to have beneficial effects on type II “fast twitch” muscle fibers and systemic inflammation, which have both been linked to losses in muscle mass and function. Unfortunately however, the findings from the limited number of factorial 2 × 2 design RCTs indicate that additional vitamin D does not enhance the effects of exercise on measures of muscle morphology, function or falls risk. However, none of these trials were adequately powered to detect a “synergistic” effect between the two treatment strategies, but it is likely that if an exercise-by-vitamin D interaction does exist, it may be limited to situations when vitamin D deficiency/insufficiency is corrected. Further targeted research in “high risk” groups is still needed to address this question, and evaluate whether there is a threshold level of serum 25(OH)D to maximize the effects of exercise on muscle and falls risk.

## 1. Introduction

One of the most striking consequences of ageing is the involuntary loss in muscle mass, a condition termed *sarcopenia.* Sarcopenia contributes to a decrease in muscle strength (force producing capacity of muscle), loss of muscle power (ability to produce force rapidly), and reduced muscular endurance (muscle fatigue) [1]. The clinical relevance of these changes to both health and functional capacity is highlighted by the increasing evidence that they have been associated with a number of metabolic, cardiovascular and musculoskeletal related disorders, particularly the decline in functional performance which has been linked to an increased risk of falls and disability, and a loss of independence and even increased mortality in the elderly [1]. Thus, identifying interventions that can prevent muscle loss and enhance muscle function to reduce the risk of falls is crucial to ensure that older adults can live independently and relatively disease and disability free into old age.

## 2. Age-Related Changes in Muscle

In both men and women the age-related loss in muscle mass begins around the fourth decade of life (~30 years of age), with the greatest losses occurring after the age of 50 [1,2]. While there is marked inter-individual variability in this loss due to differences in genetic, lifestyle and disease-related factors, it has been reported that as much as 40% of muscle mass (or size) is lost from around the age of 20 up to 60 to 90 years [1,2]. Men tend to experience a greater loss than women [3], which may be related to gender differences in hormonal factors. In addition, there is a preferential loss in muscle mass in the lower extremities [2], which has been attributed in part to the age-related reduction in physical activity. Muscle strength also peaks around the age of 30 but is maintained until about the age of 45 to 50 years [4,5,6]. Thereafter, the rate of decline in men and women is approximately 12 to 15% per decade [1,7], with even greater losses (up to 30% over 12 years) reported in some longitudinal studies [8] and the very old [9]. This reduction in muscle mass or size with ageing is due to similar losses in the number of type I (slow twitch) and type II (fast twitch) muscle fibres, but there is a selective reduction in the size of type II muscle fibres [10]. This atrophy of type II muscle is important because it may account for the accelerated loss in muscle power with ageing. For instance, it has been shown that muscle power or the ability to produce force rapidly declines earlier and more rapidly with age than muscle strength or endurance [6]. The clinical relevance of this loss in muscle power has been demonstrated in a number of studies which have reported that relative to muscle strength, the deficits in muscle power have a greater effect on functional performance [11,12]. Most activities of daily living for older adults and the elderly, such as walking, rising from a chair and climbing stairs, require power and not strength alone. Since reduced muscle power has been shown to be a strong predictor of disability [12] and falls [13] in the elderly, targeting deficits in lower extremity muscle power are likely to represent an ideal and effective strategy to reduce the risk of falls and related injuries in older adults. 

## 3. Factors Contributing to Sarcopenia

While the mechanisms responsible for the age-associated changes in muscle remain to be fully determined, multiple interrelated factors have been implicated. Decreased physical activity, poor nutrition and neurological changes have all been identified as important contributing factors [1]. Age-related reductions in serum concentrations of sex steroids, growth factors [IGF-1] and circulating 25-hydroxyvitamin D [25(OH)D] have also been implicated [1], all of which may be exacerbated by reduced levels of physical activity and inadequate nutrition. There are also indications that chronic low-grade inflammation that accompanies ageing, as reflected by increased levels of TNF-α, IL-6 and CRP [14,15] can exert a catabolic effect on muscle tissue [16]. Indeed, recent studies have reported that elevated levels of various inflammatory cytokines have been associated with sarcopenia [17], poor physical performance [18], accelerated losses in muscle strength and lean mass [17] and increased disability [19]. More specific details regarding the potential underlying mechanisms that contribute to the age-related changes in muscle can be found in several reviews [1,4]. It is clear however, that modifiable lifestyle factors can play an important role in preventing sarcopenia. In particular, regular exercise, particularly progressive resistance training, and adequate nutrition (energy, protein and vitamin D) alone or in combination are recognized as key factors that can help to prevent muscle loss and optimize muscle function to reduce the risk of falls and fractures in the elderly. 

## 4. Exercise, Muscle Health and Function in the Elderly

There is a large body of evidence from RCTs showing that progressive resistance training (PRT) can produce large increases in muscle strength (100% or more), and improve or maintain muscle mass and cross-sectional area in both older men and women [20,21,22] and even the elderly [23]. Importantly, the current evidence also indicates that there is a dose-response effect of training on muscle strength, with high intensity PRT (60–80% of maximum strength) being more effective than low-moderate intensity programs [20]. While it seems intuitive that these improvements would also enhance muscle function, these types of programs have not been shown to consistently improve measures of functional performance, including balance, gait or postural sway. Indeed, several systematic reviews have reported that conventional PRT programs have no effect on measures relating to activities of daily living and only a small to moderate effect on functional ability, despite large gains in muscle strength [24,25]. This is perhaps not surprising when considering the concept of training specificity; most traditional PRT programs encourage slow velocity muscle contractions [2–4 sec for the concentric (lifting) phase] at a moderate to high percentage of maximal force (~60–80% maximal strength). However, many common tasks related to mobility and daily perturbations require rapid coordinated and dynamic contractions within 50 to 200 ms, which is considerably less than the time needed to achieve maximal muscle force (~400–600 ms) [26]. Therefore, strategies which aim to enhance the ability to generate force quickly (muscle power), and which are specific to tasks of daily living are likely to be more relevant to the maintenance of physical function in older persons.

In recent years there has been growing interest in the role of high velocity-PRT (or power training) as a novel form of training to enhance muscle power and function in older adults. This mode of training is characterized by rapid concentric movements followed by a slower eccentric (lowering) phase performed at moderate to high loads. As highlighted in a recent meta-analysis, the findings from several RCTs conducted over 8 to 24 weeks indicate that high velocity PRT is effective for improving muscle strength, muscle power and function in healthy older adults [20]. Perhaps more importantly, this same meta-analysis reported that high velocity PRT training was more effective for improving measures of muscle function (chair rising time, stair climbing, walking speed) in older adults than traditional PRT, despite similar gains in maximal muscle strength [20]. The mechanism underlying these greater gains in function have been attributed to the fact that high velocity-PRT can improve motor-unit firing rate, synchronization, and levels of activation resulting in an increase in the number and size of type II fast twitch fibres, and thus more rapid and forceful muscle contractions which are important for dynamic functional tasks [27]. 

To our knowledge, no studies have examined whether high velocity PRT can prevent falls, but there is considerable evidence to support the role of exercise as an effective falls prevention strategy. For instance, the findings from a recent systemic review with meta-analysis of RCTs that compared fall rates in older people who undertook exercise programs compared to those that did not exercise revealed that exercise reduced the rate of falling by 17% [44 trials with 9,603 participants, rate ratio (RR) = 0.83 (95% CI, 0.75–0.91)] [28]. Furthermore, the authors reported that the greatest relative effects of exercise on fall rates were observed in those programs that incorporated high challenge balance exercises and a total dose of training >50 hours over the trial period (e.g., twice weekly over 25 weeks). Interestingly, moderate to high intensity PRT did not reduce the rate of falling and trials that included walking as part of the exercise program were associated with an increased falls risk [RR 1.32 (95% CI, 1.11, 1.58)] [28]. 

In summary, the current evidence indicates that to prevent or slow the age-related decline in muscle mass and strength, moderate to high intensity PRT should be prescribed. To help offset the decline in muscle function, high velocity PRT and targeted muscle balance and functional training appear to be most effective. For falls prevention, the available evidence indicates that older adults should undertake moderate to high challenging balance activities. However, with all training programs there is typically marked inter-individual variability in the response to training. For instance, Hubal *et al.* [29] reported that changes in muscle size and strength ranged from −2 to 59% and 0 to 250%, respectively, following 12 weeks of PRT. While there are likely to be many factors contributing to this variability, differences in nutritional status are likely to play an important role. Inadequate energy and protein intakes are recognized as two important factors that may blunt the skeletal muscle responses to training, but low serum 25-hydroyxvitamin D levels also appear to be important. The following section will briefly review the current evidence relating to the role of vitamin D on muscle function and falls risk. 

## 5. Vitamin D, Muscle Function and Falls Risk

Proximal muscle weakness has long been recognized as a predominant feature of clinical vitamin D deficiency [30]. However, with the discovery of vitamin D receptors (VDR) in muscle tissue, and the finding that their numbers decrease with age [31], there has been considerable interest into the potential therapeutic effects of vitamin D. The findings from multiple large-scale observational and prospective studies involving both men and women have shown that low serum 25(OH)D levels are related to accelerated losses in muscle mass and reduced muscular strength, reduced muscle power and gait speed, impaired balance and increased postural sway [31,32,33]. In addition, there appears to be a dose-response relationship between circulating 25(OH)D and muscle function. Data from The Third National Health and Nutrition Examination Survey (NHANES III) involving 4,100 ambulatory adults aged 60 years and older showed that measures of muscle function (8-foot walk test and repeated sit-to-stand tests) were markedly reduced in those with the lowest 25(OH)D levels (20 nmol/L), but that muscle performance tended to improve continually within the reference range of 22.5 to 95 nmol/L, with the most dramatic improvements occurring in the range of 22.5 to 40 nmol/L [31]. This association between low vitamin D and reduced muscle function has been attributed, in part, to the finding that vitamin D deficiency is associated with a selective loss and atrophy of type II muscle fibres [34], which are the fast contracting and strong muscle fibres used to avoid falling. 

**Figure 1:**
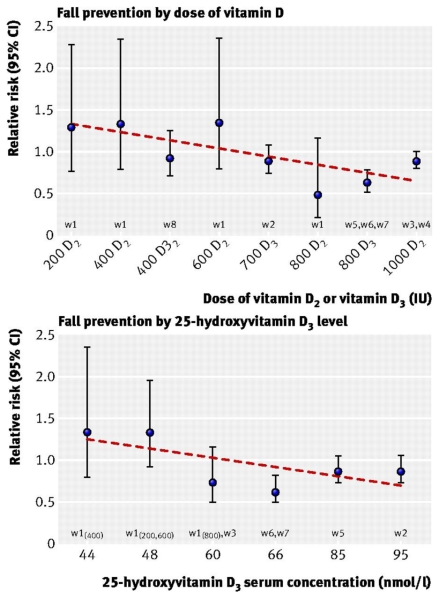
Fall prevention by dose and achieved 25(OH)D concentrations. Circles represent relative risks and error bars represent 95% confidence intervals. Trendline is based on series of effect sizes (circles). There were three trials with 800 IU D_3_^w5 w6 w7^, so the effect size for 800 IU D_3_ is the pooled result from these three trials. Likewise, the effect size for 1,000 IU D_2_ is the pooled result from the two trials with 1,000 IU D_2_^w3 w4^. We have listed the same dose D_2_ and D_3_ separately in the graph to account for their potential different impact on fall reduction. As there were two data points from the Broe *et al.* [37] trial that reached 48 nmol/L ^w1^, two trials that reached 60 nmol/L ^w1 w3^ and two trials that reached 66 nmol/L ^w6 w7^, we pooled each of the sets. On the basis of visual inspection of Figure 1, the benefits of vitamin D for fall risk started at a dose of 700 IU a day. Reproduced from Bischoff-Ferrari *et al.* [35], with permission from BMJ Publishing Group Ltd.

Over the past decade, there have been an increasing number of clinical trials which have shown that supplementation with vitamin D (or combined calcium and vitamin D) can significantly improve lower extremity muscle performance and reduce the risk of falling in the elderly (refer to Bischoff-Ferrari *et al.* [35] for an overview of high-quality trials). However, as highlighted in an earlier systematic review not all studies have observed a significant anti-fall or muscle effect of vitamin D supplementation [36]. This was largely ascribed to the use of low dose supplementation in some of these trials. In a 5-month multi-dose (200, 400, 600 or 800 IU/d), double-blind RCT in 124 nursing home residence, Broe *et al.* [37] reported that higher dose vitamin D supplementation (800 IU/d) reduced the rate of falling by 72% compared to those taking placebo or a lower dose. These findings are consistent with the results of a recent meta-analysis that included eight high quality double-blind RCTs (n = 2,426) ranging in duration from 2 months to 3 years in older individuals (mean age 65 years or older) [35]. The key findings from this study were that there was heterogeneity among trials for both the dose of vitamin D (<700 IU/d *vs.* 700–1,000 IU/d, p = 0.02) and achieved serum 25(OH)D level (<60 nmol/L *versus* ≥60 nmol/L, p = 0.005) (Figure 1). At a supplemental dose of 700–1000 IU/d, the pooled relative risk for preventing a fall was 0.81 (95% CI, 0.71–0.92), which represents a 19% risk reduction. There was no anti-fall effect for low dose supplementation (<700 IU/d, pooled RR 1.10 (95% CI, 0.89, 1.35). For an achieved circulating 25(OH)D level of ≥60 nmol/L, there was a 23% fall reduction [pooled RR 0.77 (95% CI, 0.65, 0.90)], but no effect at concentrations <60 nmol/L [pooled RR 1.35 (95% CI, 0.98, 1.84)]. Primary subgroup analysis also revealed that: (1) there were no significant differences in falls risk with trials that used vitamin D_2_ *versus* vitamin D_3_ (12% *versus* 26%, p = 0.28); (2) combined calcium plus vitamin D supplementation was not associated with a greater risk reduction; (3) there was no gender effect; (4) the benefits were present in both ambulatory and institutionalized older individuals; and (5) treatment duration did not significantly modulate the effect of vitamin D (38% fall reduction with treatment <12 months *versus* 17% with a duration ≥12 months). 

The beneficial effects of vitamin D on falls prevention have been largely attributed to the direct effect of vitamin D on muscle modulated by specific vitamin D receptors in human muscle tissue. In two prospective studies of elderly women, treatment with vitamin D (ergocalciferol) or 1-hydroxyvitamin D for 3 to 6 months was shown to increase the relative proportion and size of type II “fast twitch” muscle fibres [34,38]. However, vitamin D has also been shown to have anti-inflammatory properties, which may also explain its effects on muscle. Vitamin D deficiency has been associated with increased inflammation, and there is evidence that supplementation can suppress the release of CRP, TNF-α and IL-6 [39,40], and up-regulate the synthesis of the anti-inflammatory cytokine IL-10 [41]. Recent data also suggests that the effects of vitamin D on muscle may be mediated indirectly by lowering parathyroid hormone (PTH) [42]. High PTH levels in older community dwelling adults have been reported to be associated with increased losses in grip strength and appendicular skeletal muscle mass over 3-years [33]. In a prospective study of 637 ambulatory men and women living in institutional aged care facilities followed for a mean of 10.2 months, Sambrook *et al.* [42] reported that baseline serum PTH levels were a significant predictor of time to first fall, independent of serum 25(OH)D. Further studies are still needed to gain a greater understanding of the underlying mechanism for the association between PTH, muscle and falls. However, it is possible that the influence of PTH on falls may be related to its effect on inflammation as elevated PTH levels have been associated with increased production of IL-6 [43], which has been shown to inhibit the production of insulin-like growth factor-1 (IGF-1) and is also related to lower muscle mass and strength [44]. There is also some evidence that PTH may have a direct effect of skeletal muscle. For instance, primary hyperparathyroidism has been associated with muscle weakness that improves following parathyroidectomy [45].

In the elderly, there is some evidence that the increased rate of falling and decreased muscle strength and function may be related to the age-related decline in kidney function and the associated decrease in the production of the active form of vitamin D, 1,25-dihydroxyvitamin D [46,47,48]. Because active forms of vitamin D do not need hydroxylation in the kidney, it is hypothesized that their effects of falls should be less affected by changes in kidney function with advancing age [35]. Several RCTs have shown that treatment with 1α-hydroxyvitamin D_3_ or 1,25-dihydroxyvitamin D_3_ for 9 to 36 months can reduce the risk of falls by 16 to 24% [49,50]. While these benefits are similar to those observed with supplemental vitamin D, it is important to note that participants treated with the active forms of vitamin D were more likely to have hypercalcaemia (up to 3mmol/L) compared to the controls [35]. 

In summary, the available evidence from RCTs and meta-analyses indicate that supplemental vitamin D at a dose of at least 700–1,000 IU per day and achieving a serum 25(OH)D level of 60 to 95 nmol/L appears optimal to improve muscle function and prevent falls in the elderly. However, it is important to recognize that the dose of vitamin D required to achieve a serum 25(OH)D level of around 60 to 75 nmol/L will vary among individuals largely based on their initial 25(OH)D levels, BMI, level of sun exposure and race-ethnicity. Long-term compliance with daily supplementation is also a common problem for the elderly, but whether high dose supplementation is the answer remains to be determined in light of the recent findings from a double-blind, placebo controlled trial which found that a single annual dose of 500,000 IU administered orally to older women was associated with a significant increased risk of falls and fractures [51].

## 6. Interaction between Vitamin D and Exercise on Muscle in the Elderly

Since both vitamin D supplementation and PRT have been shown to independently have beneficial effects on muscle morphology and function in the elderly, in addition to markers of systemic inflammation which may mediate the effects, combining these two factors may represent the ideal approach to optimize muscle function and reduce the risk of falls. However, few studies have been appropriately designed to test whether additional vitamin D [or increased serum 25(OH)D] enhances the muscle or functional responses to exercise. More specifically, if there is a “synergistic interaction” between these two strategies. The term “interaction” describes the combined effects of two or more variables as being more than the sum of their individual effects. That is, an “interaction” effect implies a result which is more than simply “additive”; it is multiplicative. To test this hypothesis a factorial 2 × 2 design RCT with four groups and an adequate sample size is required: exercise and no-exercise combined with vitamin D and placebo. 

In one of the few factorial trials to have examined the potential interaction between exercise and vitamin D, Bunout *et al.* [52] reported that biweekly (~1.5 hours) strength, balance and aerobic training plus vitamin D and calcium supplementation (400 IU/d and 800 mg/d) for 9 months did not enhance muscle mass or function compared to either exercise or vitamin D alone in 96 elderly subjects aged ≥70 years with 25(OH)D <40 nmol/L. However, training significantly improved measures of muscle strength and function [timed-up-and-go (TUG)], and participants supplemented with vitamin D had greater gait speed at follow-up. The lack of an interactive effect in this trial can be attributed to a number of factors, including the small sample size, poor exercise compliance (mean 53%), the low volume and intensity of training, and the low vitamin D dose (400 IU/d) and subsequent moderate increase in serum 25(OH)D levels (31 to 66 nmol/L). 

In an 18-month factorial design RCT in 180 healthy community-dwelling men aged 50–79 years (mean 61 years), we recently reported that daily consumption of low-fat fortified milk providing an additional 800 IU vitamin D_3_, 1,000 mg calcium, 13.2 g protein and 836 kJ of energy did not significantly enhance the effects of PRT on lower extremity muscle size, strength or function [53]. Although the gains in mid-thigh muscle cross-sectional were two- to three-fold greater in the exercise plus fortified milk group compared with either of these groups alone or the control group, the “synergistic” interaction term was not significant. This was most likely due to the fact that the participants in our study were healthy community-dwelling men with adequate energy and protein intakes, and sufficient circulating 25(OH)D levels (>75 nmol/L) [53]. Based on these findings, it is likely that if an exercise-by-vitamin D (nutrition) interaction does exist, it may be limited to situations when vitamin D deficiency/insufficiency is corrected.

A recent 12-month factorial design RCT in 173 patients aged 65 years and older (mean 84 years) with acute hip fracture and vitamin D deficiency [mean 25(OH)D ~30 nmol/L] examined the combined effects of exercise and supplemental vitamin D on rate of falls [54]. In this study, participant were randomized to an extended physiotherapy (PT) exercise program (supervised 60 min/d during acute care plus an unsupervised home program) or standard PT (supervised 30 min/d during acute care with no home training), with either 2,000 or 800 IU/d of cholecalciferol (double-blinded). The primary outcome was the rate of falls, and the secondary outcome was hospital readmissions. There was no interaction between the two treatment regimens, but the authors did hypothesize *a priori* that there would be an additive and not a synergistic benefit. Subsequent main effects analysis revealed that the extended PT reduced the rate of falls by 25%, with even greater benefits observed in terms of both falls prevention and muscle function in those who participated in the home program at least once a week. Treatment with 2,000 IU/d compared to 800 IU/d of vitamin D did not lead to a greater reduction in falls, which may be explained by the finding that 25(OH)D levels increased from around 30 nmol/L to greater than 75–80 nmol/L in both groups after 12 months (800 IU/d, 88 nmol/L; 2,000 IU/d, 112 nmol/L). Interestingly however, treatment with 2,000 IU/d of vitamin D (but not extended PT) led to a 39% reduction in the rate of hospital readmissions compared to 800 IU/d. Based on these findings, the authors concluded that these two strategies may provide an ideal treatment approach because they address two different but common complications after hip fracture. 

While it is clear that further adequately powered, factorial design RCTs are needed, another important question that should be examined is whether vitamin D status can predict changes in muscle morphology or function following training. In a longitudinal study of 80 Japanese community dwelling frail elderly people, Okuno *et al.* [55] reported that the greatest improvements in various measures of functional performance (alternate step, TUG, functional reach and 5-m walk) following 3-months of training [45 min of supervised balance and mobility exercise and daily home-based exercise (walking)] occurred in those with serum 25(OH)D levels greater than 67.5 nmol/L (highest quartile). Participants with serum 25(OH)D levels below 47.5 nmol/L did not exhibit any significant improvements in function. These findings provide preliminary data indicating that low serum 25(OH)D levels may attenuate the potential exercise-induced gains in muscle function following training. However, further research is needed to resolve whether there is a threshold levels to maximize muscle health and functional responses to exercise. 

## 7. Conclusions

Regular exercise, particularly progressive resistance training, high-velocity power training and moderate- to high-challenging balance exercises, and adequate supplementation with vitamin D (at least 700–1,000 IU/d or a dose required to maintain serum 25(OH)D levels between 60 and 95 nmol/L), have been independently shown to be important for maintaining or optimizing muscle morphology, strength and power, and muscle function in older adults and the elderly. In addition, both interventions have been shown to have beneficial effects on type II muscle fibers, which are the fast contracting and strong muscle fibres used to avoid falling, as well as markers of systemic inflammation, which have been linked to sarcopenia and its detrimental correlates such as reduced muscle function, decreased muscle power, and increased disability. Therefore, combining these two modifiable lifestyle factors may represent an ideal approach to reduce the risk of falls and related injuries in the elderly. Unfortunately however, the findings from the limited number of factorial 2 × 2 design RCTs which have addressed this question indicate that additional vitamin D does not enhance the effects of exercise on measures of muscle morphology, function or falls risk. However, none of these trials were adequately powered to detect a “synergistic” effect between the two treatment strategies. Therefore, further targeted research in “high risk” groups is still needed to resolve whether supplemental vitamin D in combination with exercise can enhance muscle function, and if so, whether there is a threshold level of serum 25(OH)D to maximize the effects of exercise on both muscle performance and falls risk. 
